# Development and validation of the systemic lupus erythematous scale amongst the system of quality of life instruments for chronic diseases QLICD-SLE (V2.0)

**DOI:** 10.1186/s12955-023-02205-y

**Published:** 2023-11-29

**Authors:** Yuxi Liu, Jiaqi Zhang, Honghong Xue, Mingyang Chen, Tong Xie, Chonghua Wan

**Affiliations:** 1 School of Humanities and Management, Guangdong Medical University, Dongguan, 523808 China; 2 Institute of Health Law and Policy, Guangdong Medical University, Dongguan, 523808 China; 3 Huizhou Third People’s Hospital, Guangzhou Medical University, Huizhou, 516000 China; 4 Community Health Service Center of Liaobu Town, Dongguan, 523418 China; 5 The Affiliated Hospital of Guangdong Medical University, Zhanjiang, 524000 China; 6 Research Center for Quality of Life and Applied Psychology, Key Laboratory for Quality of Life and Psychological Assessment and Intervention, Guangdong Medical University, Dongguan, 523808 China

## Abstract

**Objective:**

The present study is aimed to develop and validate a quality of life scale for systemic lupus erythematosus (SLE) patients with Chinese cultural background, QLICD-SLE (V2.0).

**Methods:**

The QLICD-SLE (V2.0) was developed using a systematic approach that involved focus groups, nominal discussions, and pilot testing. A total of 428 SLE patients participated in the scale's assessment. Validity was examined through qualitative analysis, item domain correlation, multidimensional scaling, and factor analysis. Reliability was assessed using Pearson's correlation and Cronbach's alpha coefficients. To evaluate responsiveness, paired T-tests were conducted to compare pre- and post-treatment measurements with the standardised response mean (SRM) being calculated.

**Results:**

Correlation and factor analyses demonstrated strong construct validity. When using SF-36 as criteria, the correlation between various domains of QLICD-SLE and SF-36 ranged from 0.55 to 0.70. Test–retest correlation coefficients exceeded 0.71, and Cronbach’s alpha coefficients for both measurements in each domain were greater than or equal to 0.75. T-test results showed that both the overall score and most facet scores within each domain showed statistically significant changes after treatment (*P* < 0.05), indicating reasonable responsiveness.

**Conclusion:**

The QLICD-SLE (V2.0) appears to be a valid and reliable instrument for assessing the quality of life in patients with SLE.

**Supplementary Information:**

The online version contains supplementary material available at 10.1186/s12955-023-02205-y.

## Introduction

Systemic lupus erythematosus (SLE) is a chronic autoimmune disease with heterogeneous clinical manifestations ranging from mild cutaneous disease to catastrophic organ failure and obstetrical complications [1]. SLE predominantly occurs in young and middle-aged people with a female to male ratio of 10:1, and the kidneys and skin are the most intensively affected organs [2]. The incidence of SLE is 0.3–31.5 in 100,000 per year, and the adjusted prevalence is approaching, or even exceeding 50–100 in 100,000 [3]. Despite the progress in therapeutic options and the improvement in the survival rate, SLE remains an incurable disease [4]. SLE is characterised by immune dysregulation and aberrant production of auto-antibodies [5]. SLE is marked by a protracted course, complex and diverse clinical symptoms, and involvement of multiple organs. Once diagnosed, patients must manage the disease for an extended period. Long-term medication and recurrent flare-ups impose a significant mental and economic burden on patients, profoundly affecting their quality of life (QoL), work, and education.

When evaluating a disease's therapeutic effect, it's crucial to consider not just biological indicators for physical function but also psychological and social aspects to assess overall function (i.e. QoL).With the improvement of people’s health needs, the medical model has changed to a biological-social-psychological model, QoL has been gained more attention in medical field. QoL is a complex concept that is interpreted and defined differently within and between disciplines. In this paper, QoL refers to an individual’s perception of his or her living conditions according to the existing value and cultural system, which is related to his or her expectations and living standards [6].

Considering that the majority of Systemic Lupus Erythematosus (SLE) patients require long-term treatment, it is imperative to examine the impact of treatment on their QoL [7]. Numerous studies have explored the QoL of Systemic Lupus Erythematosus (SLE) patients, with many of them focusing on influencing factors, including disease activity, upper limb exercise, and sleep impairments. However, there are relatively few studies that have examined the instruments used to assess QoL in SLE [8–10]. While generic instruments for measuring QoL are commonly used both in the general population and among patients with Systemic Lupus Erythematosus (SLE), such as the Brief Version of World Health Organization Quality of Life [11], Short-Form-36 [12],and European Quality of Life-Dimensions [13], they often fail to capture the symptoms and side effects specific to SLE. Moreover, the variations in cultural and linguistic sensitivities among Systemic Lupus Erythematosus (SLE) patients can potentially influence their perception and reporting of QoL [14]. In contrast, disease-specific instruments like the Lupus QoL Scale (LupusQol) [15], the SLE Quality of Life Scale (L-QoL) [16] and the SLE Specific Quality of Life Scale (SLE-QoL) [17] focus on symptoms and signs that directly reflect the SLE status and are more effective than generic questionnaires. Therefore, the development of a more specific QoL measurement tailored to assess SLE-related issues would be valuable for evaluating QoL and treatment success.

Since diseases within the same disease class such as digestive diseases share many characteristics such as symptoms and side effects in common, an approach widely adopted in recent years to develop QOL instruments for diseases is to combine a general module for the entire class of diseases with the specific module for each individual disease. This approach can substantially reduce the amount of time and effort in developing new instruments, and the quality of life questionnaires from the European Organization for Research and Treatment (EORTC) and the Functional Assessment of Cancer Therapy (FACTs) have been developed based on this modular principle [18, 19]. To the best of the author's knowledge, there has been no development of a scale for Systemic Lupus Erythematosus (SLE) utilizing a modular approach that incorporates a general module in conjunction with specific modules [20]. In addition, due to cultural dependency on quality of life, there are relatively few instruments for study and application in China, and direct translations of foreign tools is not possible. For example, the family relationship and kinship play very important roles in daily life in Chinese culture. Taoism and traditional medicine focus on good temper and high spirit. Good appetite, sleep, and energy are highly regarded in daily life with food culture being very important in China. This kind of culture dependence is not reflected in most QOL instruments in other languages.

As a result, the researchers created the Chronic Disease Quality of Life Instrument (QLICD), a Qol system that contains a general module (QLICD-GM) for various diseases as well as certain disease-specific modules [21]. The most recent version of this system is QLICD (V2.0) [22], which includes 34 chronic illness specific scales, such as The Quality of Life Instruments for Chronic Diseases—Chronic Gastritis(QLICD-CG) [23] and The Quality of Life Instruments for Chronic Diseases—Chronic Obstructive Pulmonary Disease(QLICD-COPD) [24], which are widely used in some studies in China.

This research focused on developing the system’s particular module for lupus erythematosus patients, which was subsequently merged with the produced general module. This integration led to the development of the QLICD-SLE (V2.0)—a scale designed for assessing Systemic Lupus Erythematosus. The purpose of this research is to report on the scale’s development and validation.

## Methods

### Construction of the general module (QLICD-GM)

QLICD-GM (V2.0) is a generic module within the Chronic Disease Patients' Quality of Life Measurement Scale system (QLICD). It has evolved from QLICD-GM (V1.0) and encompasses three primary domains: physical function (nine items), psychological function (eleven items), and social function (eight items), totaling 28 items. Each item is rated on a five-level scale.

The development of QLICD-GM adheres rigorously to a well-established programmatic decision-making approach [25], which mainly comprises the following steps: Initially, a nominal group of 16 individuals and a focus group of 10 experts were established. The focus group consisted of 2 cardiovascular disease physicians and 9 researchers (3 quality of life/medical statistics researchers, 1 epidemiology researcher, 2 sociology researchers, and 2 psychology researchers). The nominal group consisted of 6 physicians, 2 nurses, 2 chronic disease medical educators/administrators, and 6 researchers (2 quality of life/medical statistics and epidemiology researchers, 2 sociologists, and 2 psychologists).The focus groups convened to discuss and validate the scale's structure, which encompasses three core domains: physiological, psychological, and social functioning. Secondly, after reviewing relevant literature and other famous Qol tools, such as SF-36 (Brazier et al., 1992) [26], Nottingham Health Profile (NHP) (Hunt et al., 1981) [27], QLQ-C30 (Aaronson et al., 1993) [28], combined with Chinese cultural factors, proposed the possible entries in every facet of each domain, formed 73 entries database.

**Fig. 1:**
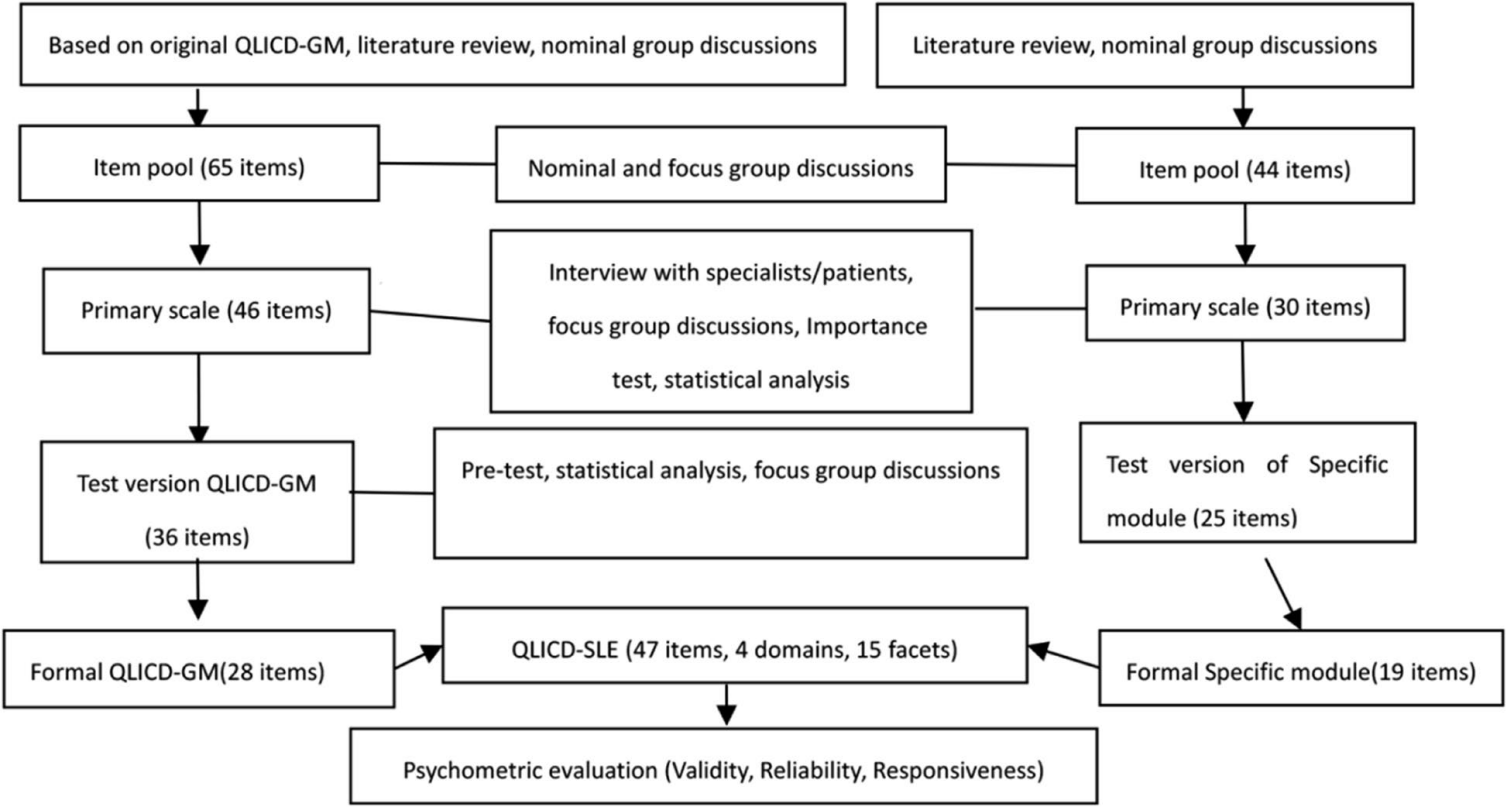
Steps towards development and validation procedure of QLICD-SLE (V2.0)

Thirdly, the focus group conducted discussions and in-depth interviews to further improve the selection of items, reducing the selected items to 46. Fourthly, 86 nominal group members and their chronic disease patients rated the importance of each item on the scale. The importance range from low to high was (0 ~ 100), and the items with low importance were deleted (average score < 65). There were 38 items in total. Four statistical methods, namely, variation analysis, comparison score standard deviation selection, correlation, factor and cluster analyses, were used to rescreen the pre-test data. Finally, 28 items were selected to form QLICD-GM, including three domains and nine facets (Fig. 1).

### Construction of the specific module (QLICD-SLE)

The nominal and focus groups used a structured decision-making process in the present study to suggest items for the specific modules of the QLICD-SLE scale and build an item pool. Firstly, the researchers chose 25 items from a library of 44 items in the SLE specific module based on a literature study, nominal group/focus group discussions, and patient interviews. Following preliminary research and two phases of screening, a module of 19 items (coded SLE1-SLE19) was developed, which included skin and mucosal symptoms (SMS), respiratory/circulation symptoms (RCS), urinary symptoms (URS), other symptoms (OTS), special mentation (SPM), and treatment side effects (TSE). See Fig. 1 in detail.

### Evaluation of QLICD-SLE

SLE patients meeting the 2019 classification criteria of the European League Against Rheumatism (EULAR) and the American College of Rheumatology (ACR) were included in the study [29]. The survey was conducted at the First Affiliated Hospital of Guagndong Medical University. 428 patients with SLE participated in the measurement of this scale. All of the individuals in this study were exclusively hospitalized patients, and most of them are adults with 20 patients aging from 13 to 17. Inclusion criteria: (1) Patients diagnosed with SLE; (2) Patients with reading, writing and expression ability and voluntary cooperation; Exclusion criteria: (1) Illiteracy; (2) Critically ill patients with other serious diseases, severe mental disorders, and confusion; (3) Patients who refused to cooperate with the investigation. The study protocol and informed consent have been approved by the Institutional Review Committee of Guangdong Medical University. All respondents participated voluntarily and provided written consent to participate (The informed consent has been obtained from their parents for study participation for minors).

The scale is composed of two parts: the general and specific modules. The general module includes three domains, namely physical function (GPH1-GPH9), psychological function (GPS1-GPS11), and social function (GSO1-GSO8), with a total of 28 items. The specific module comprised six distinct facets: skin and mucosal symptoms (SMS), respiratory/circulation symptoms (RCS), urinary symptoms (URS), other symptoms (OTS), special mentation (SPM), and treatment side effects (TSE), encompassing a total of 19 items denoted as SLE1 to SLE19.The complete scale consists of 47 items, with each item using a 5-level graded response format. A five-point equal point distance scoring method is employed to calculate item scores (ranging from 1 to 5) and the standard score for each domain, subscale, and the overall score (ranging from 0 to 100) based on standardized scoring guidelines. In the present study, two scales, QLICD-SLE (V2.0) and SF-36, were used to assess 428 SLE patients. The research team provided a brief explanation of the study's aims. Following the patients' consent, the scales were distributed to the participants, who then filled them out based on their specific circumstances. In principle, each patient completed one scale on both the first and second day of admission, as well as on the day of discharge. Two scales, QLICD-SLE and SF-36, were employed for each measurement. Subsequently, the scale's reliability, validity, and responsiveness were evaluated.

#### Content validity

The entire development process involved the active participation of clinicians, nurses, patients, quality of life researchers, and other relevant stakeholders. They convened in nominal and focus groups to engage in discussions, scrutiny, and revisions of the scale item selection, thereby ensuring the content validity of the scale.

#### Construct validity

Item-domain correlation and exploratory factor analysis (EFA) were employed to confirm the structural validity of the scale. In item-domain correlation, a Pearson's correlation coefficient (r) exceeding 0.4 indicated a strong correlation. For EFA, this study considered eigenvalues greater than 1 to assess the alignment of extracted components with the instrument's theoretical structure. Factor loadings exceeding 0.50 were applied as criteria for Varimax rotation, ensuring a clear and validated presentation of the scale's structure.

#### Criterion-related validity

The Medical Outcomes Study Short-form 36 (SF-36), developed by the American Medical Outcomes Study (MOS), is known for its high reliability and validity [30], as well as its flexibility and applicability in the Chinese population [31]. Therefore, we used the SF-36 as the gold standard to assess the correlation between corresponding domains. Pearson's correlation coefficient was employed to evaluate convergent validity, while multifaceted scaling analysis was used to examine both convergent and discriminant validity of the items. The test criteria were as follows: a correlation coefficient of ≥ 0.40 for item-domain/facet indicated convergent validity. If the item-domain/facet correlation was higher than that of other domains/facets, it demonstrated discriminant validity.

#### Internal consistency

Reliability refers to the repeatability or consistency of an item's score across assessments. In the present study, test–retest reliability (Pearson's correlation coefficient, r), internal consistency reliability (Cronbach's α), and ICC, which are commonly used indicators, were calculated for each domain/facet. Cronbach's α coefficient is a common method for evaluating internal consistency reliability in scale development. Typically, a Cronbach's α coefficient between 0.70 and 0.95 indicates good internal consistency, and test–retest reliability between 0.73 and 0.95 is considered sufficient.

#### Responsiveness

Responsiveness refers to the capacity of a scale to detect subtle yet clinically significant changes over time [32]. In this study, we calculated the average scores for each domain/facet of QLICD-SLE in both the pre-treatment and post-treatment assessments. To assess responsiveness, we employed a paired T-test and computed the Standardized Response Mean (SRM). SRM is determined by the ratio of the difference before and after treatment to the standard deviation of the difference (in absolute value). Generally, good responsiveness is indicated when the absolute SRM is > 0.8, while an SRM around 0.5 suggests moderate responsiveness, and an SRM around 0.2 indicates low responsiveness [33].

## Results

### Socio-demographic and clinical characteristics of the sample

A total of 428 SLE patients were included in this study. The majority of the study population were female (92.5 percent). In terms of ethnicity, Han individuals comprised the majority (93 percent), and the age group was primarily below 30 years old (48.4 percent). Among the patients, 269 had a fair income (62.9 percent), and most had secondary school education (37.1 percent) or a university level or higher (53.0 percent). More than half of the patients were married (62.9 percent).Regarding medical insurance, 45.1 percent of patients were self-paying, 31.3 percent had partial coverage through medical insurance, and the rest (24.1 percent) were primarily reimbursed by medical insurance. The primary occupations included professionals (9.5 percent), farmers (25.2 percent), factory workers (14.7 percent), teachers (7.0 percent), and office workers (3.5 percent). The primary course of treatment was acute cutaneous lupus erythematosus (43.5 percent), and the majority of patients were treated with hormone immunosuppressants (66.1 percent) (Table 1).

**Table 1:** Socio-demographic characteristics of the Sample (*n* = 428)

Characteristics	N	%	Characteristics	N	%
**Gender**			**Marital status**		
Male	32	7.47%	Married	269	62.85%
Female	395	92.28%	Others	159	37.15%
**Ethnic groups**			**Medical insurance**		
Han	398	92.99%	Self-paid	193	45.09%
Others	30	7.00%	Partly public insurance	134	31.31%
Missing			Public insurance	103	24.07%
**Age**			**Occupation**		
< 30	207	48.36%	Factory Worker	63	14.72%
30–39	127	29.67%	Farmer	108	25.23%
40–49	64	14.95%	Teacher	30	7.00%
50–59	20	4.67%	Office-bearer	15	3.50%
≥ 60	10	2.33%	Others	212	49.53%
Missing			**Course**		
**Income**^a^			discoid lupus erythematosus	47	10.98%
Poor	150	35.04%	Subacute cutaneous lupus erythematosus	52	12.14%
Fair	269	62.85%	acute cutaneous lupus erythematosus	186	43.45%
High	8	1.86%	Missing	143	33.41%
Missing	1	0.23%	**Treat**		
**Education**			hormone + immunosuppressant	283	66.12%
Primary school	41	9.57%	hormone	120	28.04%
High school	159	37.15%	immunosuppressant	2	0.46%
College or higher	227	53.04%	hormone immunosuppressive biologics are not used	21	4.91%
Missing	1	0.23%	Missing	2	0.46%

^a^This is evaluated by patients himself/herself according to their perceptions

### Construct validity

The correlation analysis results indicate strong associations between items and their respective domain/facet subscales (with most correlation coefficients exceeding 0.5). However, there were weaker relationships between items and other domains/facets (refer to Table 2). For instance, correlation coefficients between items GSO2-GSO8 (in bold) are higher within the same domain than across different domains. The item-dimension correlation analysis shows a strong correlation between items and the dimensions of the QLICD-SLE scale.

**Table 2:** Correlation coefficients r among items and domains of QLICD-SLE(V2.0) (*n* = 428)

Code	Items brief description	Physical	Psychological	Social	The Specific
GPH1	Appetite	0.57***	0.21	0.21	0.25
GPH2	Sleep	0.50***	0.25	0.18	0.27
GPH3	Sexual function	0.49***	0.27	0.24	0.23
GPH4	Excrement	0.43***	0.18	0.19	0.24
GPH5	Pain	0.57***	0.37	0.30	0.40
GPH6	Daily activities	0.61***	0.13	0.28	0.02
GPH7	Work	0.66***	0.29	0.46	0.12
GPH8	Walk	0.55***	0.13	0.27	0.04
GPH9	Fatigue	0.55***	0.36	0.19	0.41
GPS1	Attention	0.48	0.51***	0.49	0.32
GPS2	Memory deterioration	0.35	0.55***	0.30	0.50
GPS3	Joy of life	0.23	0.39***	0.37	0.12
GPS4	Restless	0.29	0.71***	0.36	0.40
GPS5	Family burden	0.18	0.65***	0.36	0.36
GPS6	State of health	0.17	0.66***	0.25	0.34
GPS7	Depression	0.36	0.75***	0.36	0.42
GPS8	Disappointment	0.32	0.77***	0.41	0.39
GPS9	Fear	0.17	0.70***	0.38	0.34
GPS10	Positive attitude	0.37	0.56***	0.56	0.24
GPS11	Termagancy	0.34	0.71***	0.50	0.41
GSO1	Social contact	0.45	0.47	0.70***	0.22
**GSO2**	Family relationship	0.22	0.23	0.55***	0.14
GSO3	Friend relationship	0.22	0.26	0.57***	0.17
GSO4	Family support	0.28	0.31	0.70***	0.16
GSO5	Other people’s care	0.25	0.30	0.66***	0.13
GSO6	Economic hardship	0.27	0.47	0.57***	0.34
GSO7	Labor status	0.35	0.53	0.64***	0.39
**GSO8**	Family role	0.34	0.38	0.68***	0.18
SLE1	Hair loss	0.17	0.29	0.19	0.46***
SLE2	Oral ulcer	0.24	0.20	0.13	0.45***
SLE3	Arthralgia	0.43	0.22	0.23	0.45***
SLE4	Erythema after sun exposure	0.09	0.19	0.12	0.48***
SLE5	Dry eyes and photophobia	0.30	0.41	0.21	0.67***
SLE6	Vision loss	0.23	0.34	0.25	0.61***
SLE7	Fever	0.27	0.17	0.13	0.48***
SLE8	Cough and sputum	0.30	0.25	0.20	0.53***
SLE9	Panting /be flustered	0.36	0.42	0.28	0.70***
SLE10	Chest tightness	0.23	0.25	0.26	0.60***
SLE11	Body image worse	0.12	0.34	0.22	0.60***
SLE12	Easy to catch a cold	0.32	0.36	0.27	0.59***
SLE13	Bloating and abdominal pain	0.25	0.30	0.22	0.61***
SLE14	Worry to offspring	0.05	0.33	0.14	0.48***
SLE15	Swelling of feet and eyelids	0.26	0.29	0.24	0.58***
SLE16	lumbago	0.29	0.32	0.23	0.65***
SLE17	Affect fertility	0.08	0.31	0.18	0.45***
SLE18	Unconscious	0.26	0.45	0.23	0.64***
SLE19	Migraine, refractory headache	0.27	0.36	0.19	0.63***

^***^Correlations between each item and its designated scale are in bold type

The results of the exploratory factor analysis (EFA) showed that the QLICD-GM generic module scale consists of a structural framework with 9 domains. During the factor analysis, 9 principal components were identified, contributing to a cumulative variance of 68.14 percent (see Table 3).The analysis of the specific module score for SLE revealed the extraction of 6 principal components, meeting the criterion of initial eigenvalues exceeding 1. These components contributed to a cumulative variance of 64.11 percent (see Table 4). Each of the 6 principal components corresponds to 6 facets of the specific module, including skin and mucosal symptoms (SMS), respiratory/circulation symptoms (RCS), urinary symptoms (URS), other symptoms (OTS), special mentation (SPM), and treatment side effects (TSE).

**Table 3:** Principal components and factor loadings of the general module of QLICD-SLE(V2.0) (*n* = 428)

Items	Principal components and its variance contribution (%)								
	*P1*(25.90)	*P2(*11.04)	*P3(*6.77)	*P4(*5.83)	*P5(*4.33)	*P6(*3.77)	*P7(*3.53)	*P8(*3.35)	*P9(*3.04)
GPH1							0.74		
GPH2							0.71		
GPH3									0.75
GPH4								0.75	
GPH5				0.71					
GPH6		0.86							
GPH7		0.78							
GPH8		0.80							
GPH9				0.72					
GPS1		0.50							
GPS2				0.74					
GPS3					0.54				
GPS4	0.64								
GPS5	0.55								
GPS6	0.68								
GPS7	0.77								
GPS8	0.80								
GPS9	0.77								
GPS10									
GPS11	0.56								
GSO1			0.51						
GSO2					0.82				
GSO3					0.81				
GSO4			0.82						
GSO5			0.84						
GSO6						0.79			
GSO7						0.75			
GSO8			0.64						

Factors loadings smaller than 0.5 were not displayed. Some items were not displayed because of small factor loadings

**Table 4:** Principal components and factor loadings of the specific module of QLICD-SLE(V2.0) (*n* = 428)

Items	Principal components and its variance contribution (%)					
	*P*1 (32.43)	*P*2 (8.21)	*P*3 (5.87)	*P*4 (5.55)	*P*5 (5.04)	*P6*(4.42)
SLE1						0.89
SLE2						
SLE3	0.56					
SLE4			0.75			
SLE5			0.63			
SLE6						
SLE7				0.80		
SLE8				0.60		
SLE9	0.53					
SLE10	0.52					
SLE11		0.60				
SLE12		0.58				
SLE13	0.60					
SLE14					0.83	
SLE15		0.67				
SLE16	0.69					
SLE17					0.86	
SLE18	0.67					
SLE19	0.74					

Factors loadings smaller than 0.5 were not displayed. Some items were not displayed because of small factor loadings

The first principal component included items 3, 9, 10, 13, 16, 18, and 19, primarily representing the skin and mucosal symptoms experienced by patients. This component contributed to a cumulative variance contribution rate of 25.9 percent. The second principal component comprised items 11, 12, and 15, primarily reflecting the respiratory and circulatory symptoms experienced by patients, contributing to a cumulative variance of 11.4 percent. The third principal component included major responses related to urinary symptoms in patients, specifically items 2, 4, 5, and 6.The fourth principal component mainly included items 7 and 8, reflecting other symptoms experienced by patients. The fifth and sixth principal components encompassed items 1, 14, and 17, which respectively relate to the side effects and special psychological facets of patient treatment. These six common factors collectively capture the diverse symptoms experienced by patients with SLE, aligning well with the theoretical framework and confirming the theoretical structure's good structural validity (Table 4).

### Criterion-related validity

Correlation coefficients between each domain of QLICD-SLE and the corresponding domains of SF-36 were determined. These results revealed that the correlation coefficients between the six domains of QLICD-SLE (V2.0) and the eight domains of the SF-36 scale ranged from 0.20 to 0.63. In general, a correlation coefficient greater than 0.4 is typically considered desirable. Notably, the correlation (*r* = 0.63) between the mental health function of QLC-SLE (V2.0) and the mental health function of SF-36 was higher than in the other domains, indicating robust concurrent validity (see Supplemental Table 1).

### Reliability

The reliability of the scale was evaluated by three procedures: internal consistency, test–retest and ICC (see Supplemental Table 2 for details). The data of the first measurement were used to calculate the internal consistency of each domain, and the results reveal that the Cronbach’s α of each domain and total table are greater than 0.753.The first and second test results were used to calculate the retest reliability, and the results demonstrated that test–retest correlation coefficients (r) range from 0.59 to 0.90. The retest correlation coefficients measured twice in each domain of QLICD-SLE were all greater than or equal to 0.707. Compared with the mean scores of the first and second times in each domain, there were no statistical differences in other domains except energy discomfort and respiratory and circulatory symptoms (*P* > 0.05).

### Responsiveness

To investigate reactivity, 98 patients were retested before discharge, and the paired T-test was employed to compare the mean scores before and after treatment. The results showed statistically significant differences in energy and discomfort, social function, interpersonal communication, skin and mucosal symptoms, and respiratory/circulation symptoms (*P* < 0.05).The responsiveness index SRM was used to measure mean score changes in various domains/facets of QLICD-SLE before and after treatment. SRM values for physiological, psychological, social function, and the specific module were all lower (ranging from 0.00 to 0.12) (see Supplemental Table 3).

## Discussion

The researchers systematically and efficiently developed a novel instrument system for chronic diseases known as QLICDs. This system combines a general module with a specific module tailored for individual diseases, establishing the modular approach of Disease-Qol instruments. The general module QLICD-GM can be applied to a wide range of chronic diseases. This modular approach integrates disease-specific instruments and a general module into a single scale. For example, the general module QLICD-GM can capture the overall quality of life of patients with various diseases, such as SLE and chronic gastritis (CG), while the disease-specific module QLICD-SLE captures the quality of life facets specific to SLE.

The initial phases of the study led to the development of the general module QLICD-GM, which has subsequently demonstrated reliability, validity, and responsiveness [34]. In this study, the specific module of the quality of life assessment tool designed for patients with Systemic Lupus Erythematosus (SLE) was systematically formulated. This module was constructed with a focus on various dimensions (facets), including skin and mucosal symptoms (SMS), respiratory/circulation symptoms (RCS), urinary symptoms (URS), other symptoms (OTS), special mentation (SPM), and treatment side effects (TSE). Consequently, a novel and comprehensive quality of life scale tailored to SLE patients emerged, achieved by amalgamating this specific module with the pre-existing general module known as QLICD-GM.

QLICD-SLE is the first instrument developed for quality of life in patients with systemic lupus erythematosus in China. Unlike the WHOQOL and SF-36 of two general Qol instruments, the advantage of the QLICD-SLE is that it contains disease- specific items and domains, which will provide SLE-specific information regarding patients’ perceived health status.

Furthermore, QLICD-SLE distinguishes itself from existing SLE-specific instruments, such as SLE-Qol, L-Qol, and LupusQol. SLE-Qol primarily uses a 7-point Likert scale, whereas QLICD-SLE (2.0) uses a 5-point Likert scale. While SLEQOL focuses on assessing SLE-specific Health-Related Quality of Life (HRQOL), some of its domains provide a less comprehensive assessment, leaning more towards serving as health status indicators. L-QoL, utilizing the one-parameter Rasch model, is unidimensional with good item stability and minimal Differential Item Functioning (DIF), but it falls short in providing a comprehensive measurement of specific symptoms related to SLE and cannot be used for comparing various diseases [16].

All three instruments have been validated for English-speaking patients in various cultural contexts: SLEQOL for the Singaporean Chinese population, LupusQol for predominantly White British individuals, and L-QoL for populations primarily from Northern England and London. In contrast, QLICD-SLE (2.0) was primarily developed and validated for Chinese patients with systemic lupus erythematosus.

The content of QLICD-SLE is the result of specialist expertise and patient input. It consists of a moderate number of items with a clear hierarchy (item → facet → domain → overall), allowing for score analysis at different levels, including six facets, to detect detailed changes in patients. As a result, QLICD-SLE differs from existing lupus erythematosus quality of life instrument systems.

In terms of reliability, validity, and responsiveness, a practical clinical scale should exhibit high stability, accuracy, and sensitivity [35]. The present study adheres to the World Health Organization's (WHO) definition of quality of life (WHO, 1995; WHOQOL Group, 1998) and employed pre-programmed decision-making procedures, focus group discussions, in-depth interviews, and pre-tests to construct the QLICD-SLE patient scale. These efforts effectively reduced the number of items in the final scale from an initial 65 in the universal module to 28, and from an initial 44 items in a specific module to 19, thereby preserving the scale's content validity and the integrity of its conceptual structure.

Structural validity refers to the degree of correlation between the theoretical scale structure conceived by the researcher and the scale structure established by the survey results [36]. In this research, the structural validity of the scale was primarily evaluated through item-domain correlation analysis and exploratory factor analysis (EFA). The findings show that the inter-group correlation coefficient falls between 0.1 and 0.6, while the correlation coefficient between items and their respective domains and the total item score and total scale score range from 0.3 to 0.8, indicating good reliability and responsiveness [37].

The correlation analysis in this research reveals that items in each domain have a high correlation with their respective domains but a low correlation with different domains, indicating good structural validity. Additionally, EFA was used to further assess the structural validity of QLICD-SLE. The results indicate that nine and six principal components were extracted from the 28 items of QLICD-GM and 19 items of QLICD-SLE, respectively, consistent with previous studies [38, 39]. The nine principal components reflect the nine facets of QLICD-GM within the three domains, while the six principal components correspond to six facets of the specific domain of QLICD-SLE. Therefore, the EFA results suggest that QLICD-SLE has a well-structured design.

Reliability refers to the repeatability or consistency of item scores from one assessment to another [40]. In this research, reliability was primarily assessed using retest reliability (Pearson's r), internal consistency reliability (Cronbach’s α coefficients), and ICC. In terms of retest reliability, if the health status of SLE patients remains relatively stable over a certain period, the difference in quality of life retest scores should not be statistically significant after analysis. The first measurement was conducted on the first day of admission, and the second on the second day of admission. The correlation coefficients between the two assessments for each domain reflected the consistency in the change trend of quality of life within each domain. Higher correlation coefficients indicated better retest reliability. In this research, the retest correlation coefficients (> 0.7) for all domains of the QLICD-SLE scale were high, indicating excellent retest reliability. As for internal consistency reliability, Cronbach's α coefficient ranges from 0 to 1, with higher values indicating greater scale reliability. In this study, the Cronbach's α coefficient (> 0.7) for all areas of the QLICD-SLE scale was high, signifying strong reliability.

The responsiveness of a scale refers to its ability to detect changes in patients' quality of life over time due to treatments and other factors, which should be distinguished from the scale's discriminative ability [41]. In this study, responsiveness was primarily assessed using the paired T-test for the first and second measurements (before and after treatment) across all areas of the scale, specific module facets, and the overall scale scores in SLE patients. The standardized response mean (SRM) was calculated to gauge the magnitude of effect, with values around 0.20, 0.50, and 0.80 representing small, moderate, and large responsiveness, respectively [42]. The paired T-test conducted using the QLICD-SLE scale before and after a treatment period revealed statistically significant differences in physical function, energy discomfort, social function, interpersonal communication, and urinary symptoms. This indicates a positive treatment response in these domains. However, the study found that the SRM for physiological, psychological, social function, and the specific module of the scale were all low (0.00 ~ 0.12). This may be attributed to several factors. As a chronic autoimmune disease, patients with SLE often have short hospital stays, during which they are unable to participate in regular social activities. Moreover, changes in specific modules before and after short-term treatments are not expected to be significant. Additionally, various factors can influence patients' social function, making it challenging to observe substantial changes within a brief hospitalization period.

This tool stands out as a result of its focus on Systemic Lupus Erythematosus (SLE) research conducted among non-English-speaking patients in non-English-speaking countries. More significantly, QLICD-SLE takes into account the profound influence of Chinese culture on the treatment of systemic lupus erythematosus. Chinese culture places strong emphasis on family and kinship relationships, dietary practices, temperament, and spirituality. QLICD-SLE delves into various facets of this cultural heritage, including appetite, sleep, energy, and family support.

Several limitations are noteworthy in this study. Firstly, the subjects were exclusively selected from hospital inpatients, which potentially introduce selection bias. Secondly, while the QLICD-SLE(V2.0) scale has been developed and evaluated within the context of Chinese culture and population, further investigations are necessary to assess its applicability among outpatients in local clinics and patients from other East Asian countries. Lastly, it's important to acknowledge that the assessment of the quality of life in SLE patients relied on conventional psychometric principles rather than clinimetric criteria [43], underscoring the imperative for future research dedicated to evaluating the clinimetric properties of this rating scale [44].

## Conclusion

The present study developed QLICD-SLE (V2.0) specifically tailored for the Chinese population, considering the influence of national culture on the assessment of quality of life. This tool aligns with Chinese culture and utilizes a hierarchical structure, comprising the overall scale, domains, facets, and items, to facilitate comprehensive health assessment. The study involved 428 SLE patients, demonstrating good responsiveness, validity, and reliability. It has promising future for diverse clinical applications, including but not limited to assessing the impact of various treatment modalities on the quality of life in Systemic Lupus Erythematosus (SLE) patients, facilitating personalized patient management plans, and serving as a valuable tool for evaluating the effectiveness of interventions in clinical research studies.

### Supplementary Information

**Additional file 1:** **Supplemental Table 1.  **Correlation coefficients among domains scores of QLICD-SLE(V2.0) and SF-36  (*n*=428). **Supplemental Table 2  **Reliability, floor and ceiling effects of the quality of life instrument QLICD-SLE(V2.0)  (*n*=428 for α, and floor and ceiling effects, n= 73  for r, ICC). **Supplemental Table 3  **Responsiveness of the quality of life instrument QLICD-SLE(V2.0)  (*n*=428). 

**Additional file 2: Supplemental Table 1.  **Correlation coefficients among domains scores of QLICD-SLE(V2.0) and SF-36  (*n*=428). **Supplemental Table 2.  **Reliability, floor and ceiling effects of the quality of life instrument QLICD-SLE(V2.0)  (*n*=428 for α, and floor and ceiling effects, *n*= 73  for r, ICC). **Supplemental Table 3  **Responsiveness of the quality of life instrument QLICD-SLE(V2.0)  (*n*=428). 

## Data Availability

The datasets used and analyzed during the current study are available from the corresponding author on reasonable request.
