# Supplementary material for: Development and validation of the systemic lupus erythematous scale amongst the system of quality of life instruments for chronic diseases QLICD-SLE (V2.0)
Source: Health Qual Life Outcomes. 2023 Nov 29;21:128. doi: 10.1186/s12955-023-02205-y (PMC10685541; doi:10.1186/s12955-023-02205-y)
Supplement: Supplementary file 1 — Additional file 1: Supplemental Table 1. Correlation coefficients among domains scores of QLICD-SLE(V2.0) and SF-36 (n=428). Supplemental Table 2 Reliability, floor and ceiling effects of the quality of life instrument QLICD-SLE(V2.0) (n=428 for α, and floor and ceiling effects, n= 73 for r, ICC). Supplemental Table 3 Responsiveness of the quality of life instrument QLICD-SLE(V2.0) (n=428). [file 12955_2023_2205_MOESM1_ESM.docx]

**Supplemental Table 1** Correlation coefficients among domains scores of QLICD-SLE(V2.0) and SF-36 (n=428)

| **QLICD-SLE** | **SF-36** | | | | | | | | |
| --- | --- | --- | --- | --- | --- | --- | --- | --- | --- |
|  | **Physical function** | **Role-**  **physical** | **Body pain** | **General**  **health** | **Vitality** | | **Social**  **function** | **Role-**  **emotional** | **Mental**  **health** |
| **Physical** | **0.52** | 0.46 | **0.58** | 0.54 | 0.59 | 0.50 | | 0.47 | 0.43 |
| **Psychological** | 0.28 | 0.34 | 0.31 | 0.46 | **0.58** | 0.46 | | 0.45 | **0.63** |
| **Social** | 0.35 | 0.39 | 0.37 | 0.46 | 0.52 | 0.49 | | 0.43 | 0.52 |
| **The specific** | 0.20 | 0.27 | 0.37 | 0.39 | 0.46 | 0.37 | | 0.34 | 0.39 |

**Supplemental Table 2** Reliability, floor and ceiling effects of the quality of life instrument QLICD-SLE(V2.0) (n=428 for α, and floor and ceiling effects, n= 73 for r, ICC)

| 9Domains/facets | Internal consistency  Coefficient α | Test-retest reliability correlation *r* | ICC (95%CI) | Floor and ceiling effects (%) |
| --- | --- | --- | --- | --- |
| **Physical domain (PHD)** | **0.70** | **0.74** | **0.74(0.60-0.85)** | **0.00, 0.00** |
| Basic physiologic functions (BPF) | 0.49 | 0.76 | 0.76(0.55-0.88) | 0.53, 0.72 |
| Independence (IND) | 0.55 | 0.59 | 0.59(0.33-0.79) | 0.71, 48.80 |
| Energy and discomfort (EAD) | 0.15 | 0.70 | 0.70(0.56-0.81) | 2.33, 2.65 |
| **Psychological domain (PSD)** | **0.85** | **0.83** | **0.83(0.71-0.90)** | **0.72, 0.53** |
| Cognition (COG) | 0.43 | 0.78 | 0.78(0.67-0.86) | 0.52, 7.74 |
| Emotion (EMO) | 0.82 | 0.77 | 0.77(0.63-0.86) | 0.51, 0.73 |
| Will and personality (WIP) | 0.48 | 0.66 | 0.66(0.46-0.82) | 0.50, 12.6 |
| **Social domain (SOD)** | **0.77** | **0.81** | **0.81(0.66-0.91)** | **0.50, 1.62** |
| Interpersonal communication (INC) | 0.63 | 0.72 | 0.72(0.61-0.85) | 0.51, 19.22 |
| Social support and security (SSS) | 0.54 | 0.77 | 0.77(0.62-0.88) | 0.22, 3.70 |
| Social role (SOR) | 0.39 | 0.59 | 0.59(0.34-0.81) | 0.71, 12.10 |
| **Sub-total (QLICD-GM)** | **0.86** | **0.85** | **0.85(0.72-0.93)** | **0.23, 0.53** |
| **Specific domain (SPD)** | **0.87** | **0.88** | **0.88(0.75-0.95)** | **0.23, 0.23** |
| Skin and mucosal symptoms(SMS) | 0.42 | 0.74 | 0.74(0.60-0.84) | 0.52, 4.24 |
| Respiratory/circulation symptoms (RCS) | 0.63 | 0.71 | 0.71(0.51-0.86) | 0.22, 7.94 |
| Urinary symptoms(URS) | 0.60 | 0.78 | 0.78(0.60-0.88) | 1.23, 16.60 |
| Other symptoms(OTS) | 0.65 | 0.84 | 0.84(0.72-0.92) | 1.43, 4.44 |
| Special mentation(SPM) | 0.52 | 0.87 | 0.87(0.79-0.93) | 22.01,9.82 |
| Treatment side effects(TSE) | 0.56 | 0.80 | 0.80(0.60-0.92) | 0.91, 8.43 |
| **Total (TOT)** | **0.92** | **0.90** | **0.90(0.82-0.95)** | **0.22, 0.21** |

ICC: intra-class correlation, CI: confidence interval.

**Supplemental Table 3** Responsiveness of the quality of life instrument QLICD-SLE(V2.0) (n=428)

| Domains/facets | Before treatment  Mean SD | | After treatment  Mean SD | | Differences  Mean SD | | | *t* | *p* | SRM |
| --- | --- | --- | --- | --- | --- | --- | --- | --- | --- | --- |
| **Physical domain** | **66.62** | **13.41** | **67.72** | **12.40** | | **1.43** | **11.93** | **-1.907** | **0.058** | **0.09** |
| Basic physiologic functions | 57.51 | 14.42 | 57.51 | 14.41 | | 0.04 | 13.22 | -0.057 | 0.955 | 0.00 |
| Independence | 85.54 | 20.91 | 86.13 | 19.52 | | 1.53 | 18.01 | -1.416 | 0.158 | 0.03 |
| Energy and discomfort | 56.33 | 23.22 | 60.64 | 22.23 | | 3.82 | 23.21 | -2.705 | 0.007 | 0.19 |
| **Psychological domain** | **60.10** | **18.54** | **60.13** | **18.21** | | **-0.91** | **14.32** | **1.052** | **0.293** | **0.00** |
| Cognition | 65.12 | 22.32 | 64.21 | 21.82 | | -1.83 | 19.34 | 1.554 | 0.121 | 0.05 |
| Emotion | 56.73 | 20.70 | 57.13 | 20.53 | | -0.52 | 17.03 | 0.53 | 0.597 | 0.02 |
| Will and personality | 67.32 | 22.94 | 66.64 | 22.32 | | -1.30 | 21.11 | 1.027 | 0.305 | 0.03 |
| **Social domain** | **72.11** | **16.22** | **70.73** | **17.41** | | **-1.73** | **13.20** | **2.154** | **0.032** | **0.11** |
| Interpersonal communication | 78.32 | 17.33 | 76.52 | 17.33 | | -2.92 | 14.92 | 3.276 | 0.001 | 0.12 |
| Social support and security | 69.74 | 19.01 | 67.61 | 21.32 | | -1.71 | 17.61 | 1.614 | 0.108 | 0.12 |
| Social role | 66.32 | 23.52 | 66.70 | 23.24 | | 0.12 | 21.53 | -0.104 | 0.917 | 0.02 |
| **Sub-total (QLICD-GM)** | 65.60 | 13.53 | 65.62 | 13.72 | | -0.43 | 10.42 | 0.65 | 0.516 | 0.00 |
| **Specific domain** | **68.03** | **16.74** | **69.41** | **16.81** | | **1.64** | **12.11** | **-2.265** | **0.024** | **0.12** |
| Skin and mucosal symptoms | 67.31 | 20.42 | 69.62 | 19.53 | | 2.65 | 18.03 | -2.444 | 0.015 | 0.13 |
| Respiratory/circulation symptoms | 74.62 | 20.31 | 76.83 | 19.04 | | 2.73 | 18.64 | -2.448 | 0.015 | 0.12 |
| Urinary symptoms | 71.82 | 23.60 | 72.84 | 24.20 | | 1.10 | 21.82 | -0.823 | 0.411 | 0.05 |
| Other symptoms | 68.83 | 20.24 | 69.93 | 21.43 | | 0.43 | 15.53 | -0.407 | 0.685 | 0.07 |
| Special mentation | 47.81 | 34.32 | 49.12 | 34.62 | | 3.02 | 25.41 | -1.973 | 0.049 | 0.05 |
| Treatment side effects | 69.52 | 22.81 | 69.81 | 21.71 | | 0.83 | 19.42 | -0.695 | 0.488 | 0.02 |
| **Total (TOT)** | **66.61** | **13.10** | **67.12** | **13.43** | | **0.41** | **9.31** | **-0.763** | **0.446** | **0.05** |
